# Revisiting NO_2_ as Protecting Group of Arginine in Solid-Phase Peptide Synthesis

**DOI:** 10.3390/ijms21124464

**Published:** 2020-06-23

**Authors:** Mahama Alhassan, Ashish Kumar, John Lopez, Fernando Albericio, Beatriz G. de la Torre

**Affiliations:** 1 Peptide Science Laboratory, School of Chemistry and Physics, University of KwaZulu-Natal, Durban 4001, South Africa; 214584820@stu.ukzn.ac.za (M.A.); KumarA1@ukzn.ac.za (A.K.); 2 KwaZulu-Natal Research Innovation and Sequencing Platform (KRISP), School of Laboratory Medicine and Medical Sciences, College of Health Sciences, University of KwaZulu-Natal, Durban 4041, South Africa; 3 Novartis Pharma AG, Lichtstrasse 35, CH-4056 Basel, Switzerland; john.lopez@novartis.com; 4 CIBER-BBN, Networking Centre on Bioengineering, Biomaterials and Nanomedicine, 08028 Barcelona, Spain; 5 Department of Organic Chemistry, University of Barcelona, 08028 Barcelona, Spain; 6 Institute for Advanced Chemistry of Catalonia (IQAC-CSIC), Jordi Girona 18-26, 08034 Barcelona, Spain

**Keywords:** δ-lactam formation, microwave, NBP, orthogonal protection, protecting group, sonochemistry, side-reaction, solid-phase peptide synthesis, ultrasounds

## Abstract

The protection of side-chain arginine in solid-phase peptide synthesis requires attention since current protecting groups have several drawbacks. Herein, the NO_2_ group, which is scarcely used, has been revisited. This work shows that it prevents the formation of δ-lactam, the most severe side-reaction during the incorporation of Arg. Moreover, it is stable in solution for long periods and can be removed in an easy-to-understand manner. Thus, this protecting group can be removed while the protected peptide is still anchored to the resin, with SnCl_2_ as reducing agent in mild acid conditions using 2-MeTHF as solvent at 55 °C. Furthermore, we demonstrate that sonochemistry can facilitate the removal of NO_2_ from multiple Arg-containing peptides.

## 1. Introduction

Arginine (Arg) is a key natural amino acid that is found in many biologically active peptides [1]. Its characteristic structural feature is the presence of a guanidino group in its side-chain. This group is strongly basic, with a *pK_a_* value of 12.5, which means the side-chain remains protonated under physiological conditions [2,3]. This guanidino moiety is key for the biological activity of Arg-containing peptides [4]. In this regard, several active pharmaceutical ingredients (APIs) contain one or even several Arg residues.

For instance, this intriguing amino acid is found in desmopressin, leuprolide, and the more recently approved abaloparatide, angiotensin II, bremelanotide and afamelanotide from the α-melanocyte-stimulating hormone (α-MSH), semaglutide and other drugs approved earlier from the glucagon-like peptide family (GLP) [5]. It is important to highlight the case of etelcalcetide, which was approved by the FDA in 2017 for the treatment of secondary hyperparathyroidism in adults with chronic kidney disease on hemodialysis. It is a simple octapeptide formed by a linear chain of seven D-amino acids (one D-Cys, one D-Ala, and five D-Arg) linked to an L-Cys through a disulphide bridge [6,7]. Multiple Arg residues are also very common in antimicrobial peptides (AMPs) and the so-called cell-penetrating peptides (CPPs) [8,9,10]. In fact, the unique characteristic of most CPPs is the presence of several Arg residues, as exemplified in the fragment 48–60 of the transactivator of transcription (TAT) of HIV and octa-Arg [11].

The majority of peptides used for research and industrial purposes are prepared by a chemical process, the fluorenylmethoxycarbonyl (Fmoc)/*tert-*butyl (tBu) Solid-Phase Peptide Synthesis (SPPS) approach being the most convenient [12]. Although the protonated guanidino group of Arg should show poor reactivity in front of acylation, which is the key reaction in peptide synthesis, it has to be protected to facilitate its solubility in the common solvents used in SPPS and, more importantly, to avoid the two possible side-reactions. On one hand, some Orn can be formed during the stepwise peptide chain elongation or during the cleavage [13,14], and on the other hand, the most important side-reaction, the δ-lactam formation, is a process driven by the six-member ring formed [15]. δ-Lactam formation takes place during the coupling step, when the carboxylic group of Arg is activated. In contrast to other side-reactions, this cyclic amide is not directly converted into an impurity in the target peptide. Instead, it causes an impractical consumption of the activated Arg, which in turn can be indirectly translated into incomplete incorporation of the Arg and therefore the presence of a deletion (des-Arg) peptide. To mitigate this, repetitive couplings of protected Arg are carried out, a protocol that increases the cost of the whole synthetic process. This drawback is exacerbated during the industrial preparation of Arg-containing peptides, where minimum excesses of reagents are used and repetitive couplings imply high solvent and reagent consumption and a longer process time, and therefore a greater overall production cost. Of note, in an industrial mode, the most currently used Arg derivative (Fmoc-Arg(Pbf)-OH, Pbf for 2,2,4,6,7-pentamethyldihydrobenzofuran-5-sulfonyl, see below) is the most expensive of all protected proteinogenic amino acids. Thus, in a 100-g scale, the cost of Fmoc-Arg(Pbf)-OH is approximately 10 times more than Fmoc-Phe-OH (€360 vs. €36). Furthermore, it is important to consider atom economy, which is 0.27 for the former and 0.40 for the latter. These figures mean that the synthesis of a peptide containing Arg is approximately 15 times more expensive in comparison with those containing Phe or other amino acids. In this scenario, approaches to optimize Arg protection, coupling, and protecting group removal could have an important impact in the industrial sector.

**Figure 1:**
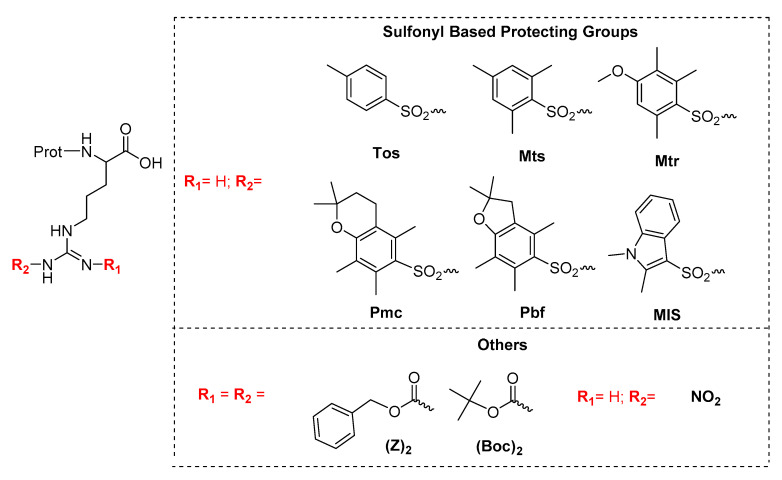
Most used Arg side-chain protecting groups.

The protection of Arg in the Fmoc strategy has its roots in *tert-*butoxycarbonyl (Boc) chemistry, where Arg is protected with the tosyl (Tos) group, which is removed by anhydrous HF, trifluoromethanesulfonic (TFMSA) or related acids [16]. Thus, Yajima’s group developed mesityl-2-sulfonyl (Mts) [17], and later Masahiko et al., after the in-depth study of several candidates, developed the more labile 4-methoxy-2,3,6-trimethylphenylsulfonyl (Mtr) [18], which is removed with almost neat trifluoroacetic acid (TFA) in the presence of scavengers over a prolonged period. An important breakthrough in this field was the development of the 2,2,5,7,8-pentamethylchroman-6-sulfonyl group (Pmc) by Ramage and co-workers [19,20]. The structure of this group recasts the methoxy in position 4 on Mtr in a cyclic ether and keeps three methyl groups on the aryl moiety. Pmc is more labile than Mtr. Carpino and co-workers went a step further when they discovered that the five-member ring present in the Pbf makes this group more labile than the six-member ring of the Pmc [21]. Verdini et al. [22] developed the bis-Boc protection, blocking both N^ω^ and N^ω’^ for the guanidino group of Arg. Both Boc groups are removed by TFA-H_2_O (95:5) at room temperature (rt) in 1 h. Although our group has reported that 1,2-dimethylindole-3-sulfonyl (MIS) is much more labile than the Pbf group [23], the latter continues to be the most widely used protecting group for the side-chain of Arg. However, in addition to its high price, which is mainly due to an extremely difficult production process, Pbf is not exempt from the side-reactions outlined earlier, such as δ-lactam formation as has been recently reported by our group [24] (see Figure 1 for the structures of these protecting groups).

In the context of SPPS, little attention has been paid to the use of the NO_2_ group, which was introduced by Bergmann et al. [25] at the beginning of the era of protecting groups and has been applied mostly in solution chemistry. The strong electron-withdrawing character of the NO_2_ group reduces the basic nature of the guanidino group, thereby modulating its reactivity. Catalytic hydrogenation using a wide range of catalysts such Pd black, [26] SnCl_2_, [27] and TiCl_3_ [28] has been proposed for the removal of the NO_2_ group. Catalytic hydrogenation is only friendly used in organic chemistry laboratories, but not in those devoted to other biosciences. Furthermore, this reaction works relatively without difficulty for shorter peptides containing a single Arg(NO_2_) residue. However, with multiple Arg(NO_2_) residues, catalytic hydrogenation can lead to difficulties, which include a long reaction time with the concomitant degradation of the target peptide [29]. Conversion of a Arg(NO_2_) residue into an aminoguanidino-derivative [30] and reduction of the aromatic ring of a Trp and even Phe residue [31] have been observed in some cases.

Here we revisit the use of the NO_2_ group for Arg protection in SPPS. In this regard, we first compared the stability of the NO_2_ derivative of Arg with the Pbf and (Boc)_2_ derivatives in *N,N-*dimethylformamide (DMF) and the green solvent *N-*butylpyrrolidone (NBP), as well as their tendency to render δ-lactam formation in both solvents [32]. We then developed a method for removing the NO_2_ group without the concourse of catalytic hydrogenation. 

## 2. Results and Discussion

### 2.1. Stability of Fmoc-Arg(x)-OH in Solution

The stability of three Fmoc-Arg(X)-OH analogues [X = (Boc)_2_, NO_2_, and Pbf] was studied in solution over a period of time. Solutions (0.2 M) of each analogue in both DMF and NBP were prepared in closed high-performance liquid chromatography (HPLC) vials following the procedure described previously by our group [24]. The reaction was monitored by reverse phase HPLC (Figures S1–S6).

Fmoc-Arg(Boc)_2_-OH slowly degraded in both solvents over time, rendering mainly the mono protected Fmoc-Arg(Boc)_2_-OH, which was already present in the commercial sample (Table 1). Nevertheless, solutions of Fmoc-Arg(Boc)_2_-OH in the two solvents could be kept up to one week, thereby making it compatible with peptide synthesizers and also for industrial manufacturing where the solution is prepared at the time of coupling. For longer periods, the degradation was slightly higher in NBP. In contrast, the NO_2_ and Pbf analogues were totally stable.

**Figure 2:**
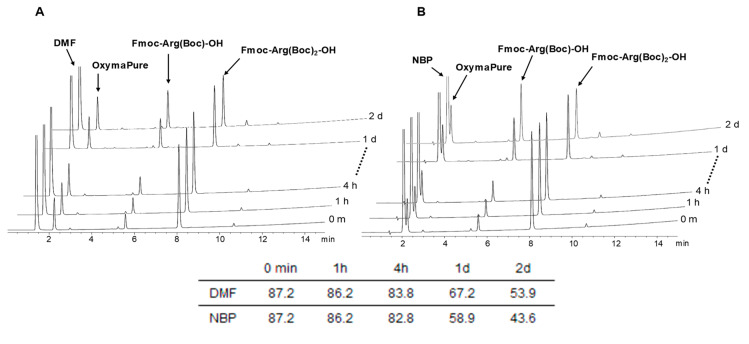
Stability of the mixture of Fmoc-Arg(Boc)_2_-OH/OxymaPure 1:1 in DMF (**A**) and NBP (**B**) at 45 °C. Elution: 30-95% of B into A in 15 min.

Stability was examined again at 45 °C in DMF and NBP, in the presence of OxymaPure, which are conditions commonly used in SPPS. Again, Pbf and NO_2_ analogues showed total stability (Figures S7–S10), while the bis Boc derivative degraded slightly faster than in the previous experiment, but could still be considered compatible with the standard reaction times for coupling (up to 4 h). As in the previous case, the degree of degradation was higher in NBP than DMF over a longer period (Figure 2).

### 2.2. δ-Lactam Formation

**Scheme 1:**
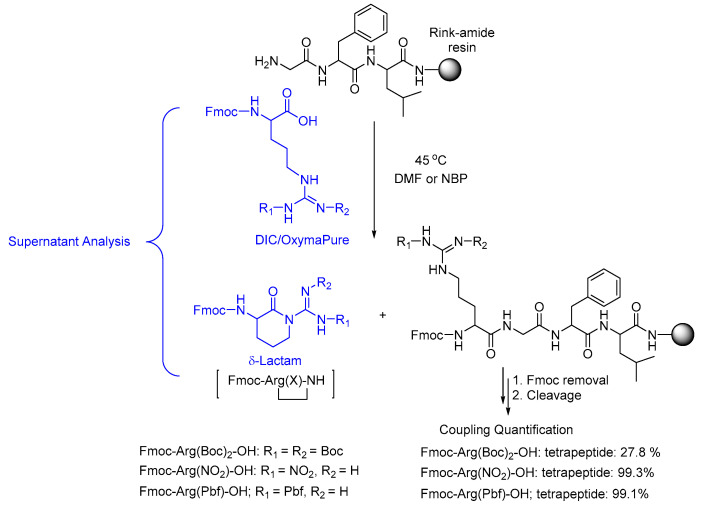
Activation of protected Arg derivatives and incorporation on a tripeptidyl resin.

The formation of δ-lactam was studied following Scheme 1. The carboxylic groups of the three protected Arg derivatives were activated using *N,N’-*diisopropylcarbodiimide (DIC) and OxymaPure as additive in an equimolar mixture (1:1:1) in DMF or NBP at 45 °C. The mixture (1.5 equiv.) was then added onto the peptide H-Gly-Phe-Leu-NH-Rink-amide-polystyrene-resin. Aliquots of the supernatant were taken at different times and analyzed by HPLC. After 120 min of coupling, the resin was filtered, washed and Fmoc removed before cleavage and total deprotection (except in case of the NO_2_ group) using high TFA-triisopropylsilane (TIS)-H_2_O (95:2.5:2.5). The crude peptides were then analyzed by HPLC [24].

**Figure 3:**
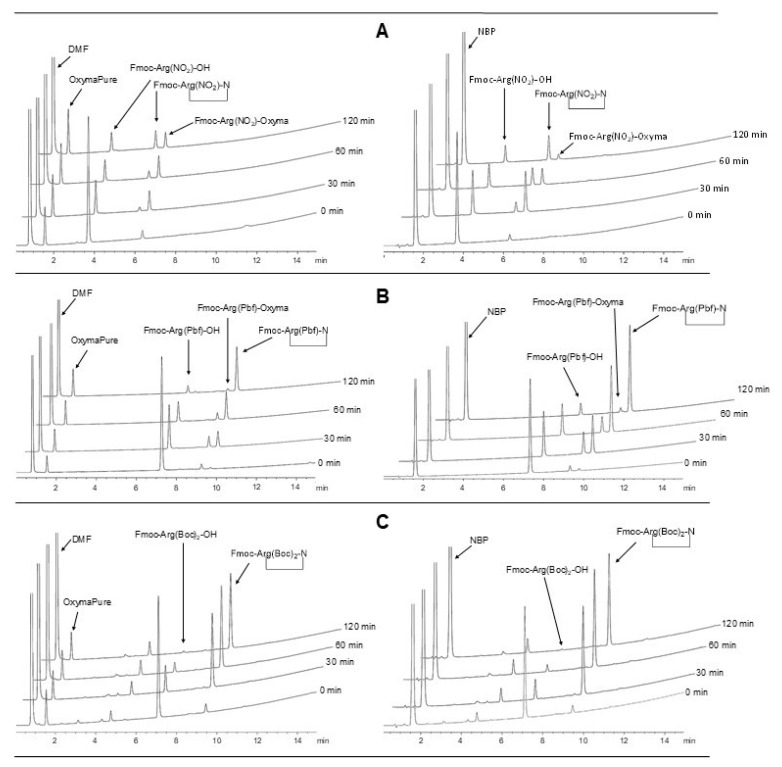
High-performance liquid chromatography (HPLC) chromatograms of the supernatants after the protected Arg derivatives [**A**, NO_2_; **B**, Pbf; **C**, (Boc)_2_] were activated with DIC (*N,N’-*diisopropylcarbodiimide) and OxymaPure and then added to the peptidyl resin. Elution: 30–95% of B into A in 15 min.

The analysis of the supernatants of each derivative revealed that DMF and NBP showed a similar behavior in the three protecting groups. More importantly, the same analysis showed that Fmoc-Arg(NO_2_)-OH had the least tendency to form δ-lactam. There was a good fertile consumption (the decrease in protected Arg was not translated into the formation of δ-lactam), (In the context of this manuscript, “fertile consumption” refers to the decrease in the presence of the protected Arg derivative when it remains as an active ester after activation or acylates the peptide resin) because at 30 min the 75% decrease in Fmoc-Arg(NO_2_)-OH (It is also important to consider that part of the protected Arg shown after 30 min could derive from the hydrolysis of the corresponding active ester and therefore would be part of the “fertile consumption”.) was translated into only 3% of δ-lactam, having approximately 15% of active ester (Figure 3A). This finding implies that the rest of the monomer was incorporated into the peptidyl resin, which was corroborated by high coupling (>99% yield). For Fmoc-Arg(Pbf)-OH, the kinetics was slower, except for δ-lactam formation (Figure 3B). Thus, at 30 min, the formation of δ-lactam was greater (12%, four times more) than for the NO_2_ derivative. Furthermore, although the fertile consumption of the initial Fmoc-Arg(Pbf)-OH (40% decrease of the initial protected Arg^2^ and 8% formation of the active ester) was less than that achieved by the NO_2_ analogue, the result was that after 120 min it yielded same coupling efficiency (>99%). Finally, Fmoc-Arg(Boc)_2_-OH showed the fastest kinetics of δ-lactam formation (60%) (Figure 3C), which translated into low coupling efficiency (28%) as a result of the side-reaction. Overall, the lower tendency of the NO_2_ analogue vs. the (Boc)_2_ and Pbf analogues to render δ-lactam is reflected by the observation that the important increment of the side-product occurred after 60 min in NO_2_, when presumably the coupling had been completed and therefore the active ester could no longer be fertile. Even at 120 min, a ratio of almost 1:1 of δ-lactam and active ester was present. In the case of Pbf analogue at 120 min, almost all the active ester had been converted to the side-product. Finally, in the case of the (Boc)_2_ analogue, the active ester was not present at any time, only the δ-lactam was found.

### 2.3. On-Resin Removal of NO_2_ from the Arg Side-Chain

**Figure 4:**
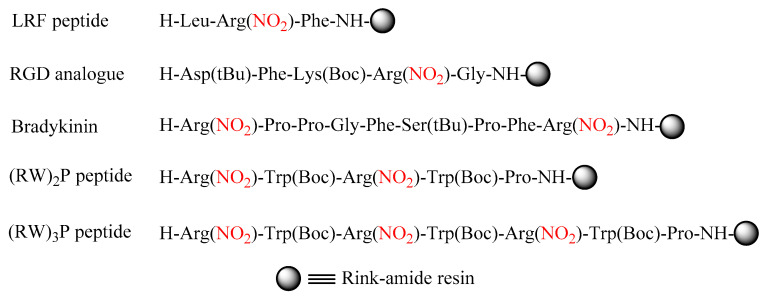
Peptidyl resins used in this study.

Once the suitability of the NO_2_ derivative of the Arg had been demonstrated in terms of stability and the low tendency of δ-lactam formation, our attention turned to the development of a new removal method with broad applicability and easy handling. Inspired by our previous work on the removal of *p*-nitrobenzyloxycarbonyl (pNZ) as protecting group of amines in SPPS [33], the capacity of SnCl_2_ in a slightly acid medium to remove the NO_2_ group was investigated. A similar method has been extensively used in the early times of combinatorial chemistry to reduce polymer-supported nitro aromatic compounds [34,35]. Taking the tripeptide H-Leu-Arg(NO_2_)-Phe-NH-Rink-amide-resin as a model, an initial fine-tune of the removal strategy was carried out. The conditions were then further adjusted with the peptidyl resins shown in Figure 4. The list included the lineal precursor of Cilengitide and bradykinin, two peptides of biological interest, and also two peptides containing the Arg-Trp sequence repeated twice and three times. These molecules thus allowed us to study the effect of an increasing number of NO_2_ groups to be removed, as well as the effect of the removal conditions on a fragile residue like Trp. As references, the same peptides were synthesized using Fmoc-Arg(Pbf)-OH. All peptides synthesized with NO_2_- or Pbf-protecting groups were cleaved from the resin using TFA-TIS-H_2_O (95:2.5:2.5) for 1 h at r.t.

**Figure 5:**
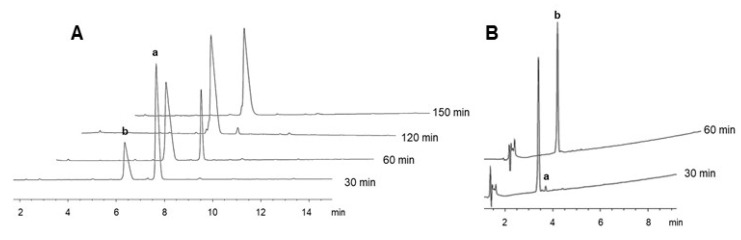
HPLC of the removal of the NO_2_ group from H-Leu-Arg(NO_2_)-Phe-NH-Rinkamide-resin (**A**) and H-Asp(OtBu)-Phe-Gly-Arg(NO_2_)-Gly-NH-Rink-amide-resin (**B**) using 2 M SnCl_2_-0.04 M phenol-0.2 M aq HCl in 2-MeTHF at 55 °C. **a:** protected peptide; **b:** unprotected peptide. Elution: 10-25% of B into A for (**A**) and 5-95% of B into A for (**B**) in 15 min.

As a starting point, using the H-Leu-Arg(NO_2_)-Phe-NH-Rink-amide-resin as substrate and the conditions developed by our group to remove pNZ (solutions of 6–8 M SnCl_2,_ 0.4 M phenol, either 0.064 M HCl-dioxane or 0.0016 M AcOH in DMF at 55 °C), the following parameters were studied: concentration of SnCl_2_ (taking into consideration that 8 M SnCl_2_ is a supersaturate solution of difficult handling), the need of phenol, the best acid rectifier and its concentration, the temperature, and the solvent (SI, Table 1). The results can be summarized as follows: (i) after assaying DMF, DCM/DMF, and the green solvents MeOH, EtOH, NBP, cyclopentylmethyl ether (CPME), and 2-methyltetrahydrofurane (2Me-THF), the best results were found with the latter. While DMF gave acceptable results, NBP was, on this occasion, less suitable. The use of EtOH gave slower kinetics and MeOH and CPME yielded the poorest results, probably due to its poor capacity to swell the resin; (ii) 2 M SnCl_2_ rendered similar (or even better) performance than 6-8 M solutions and therefore is the concentration preferred; (iii) the absence of phenol gave slightly poorer results; and (iv) the use of 0.2 M aq HCl, which is easier to use, proved to be good substitute for HCl-dioxane. This final concentration of acid is compatible with the presence of acid-labile protecting groups and it does not cause cleavage from the resin since it represents only 0.64% of the solution. Additionally, in some of these experiments, it was observed that the kinetics during the first hour were very slow, and that the reaction was faster after addition of fresh solution. Therefore, a previous washing with a solution of 0.2 M aq HCl in 2-MeTHF was carried out before the removal treatment. This approach greatly enhanced the removal. This acidic washing can neutralize traces of the piperidine used to remove the Fmoc group at the end of the peptide chain elongation. As a result of this preliminary set of experiments, washings with 0.2 M aq HCl in 2-MeTHF (3 × 1 min) followed by 2 M SnCl_2_-0.04 M phenol-0.2 M aq HCl in 2-MeTHF (2–3 × 1 h) at 55 °C were considered optimal conditions for the LRF model sequence (Figure 5A). These conditions were then applied successfully to the RGD peptides (Figure 5B). Interestingly, the removal kinetics for the RGD pentapeptide were much faster than for the LRF tripeptide. After a single treatment of 30 min, the RGD peptide showed more than 97% deprotection whilst only 30% was observed in case of LRF. This finding prompted us to do a new assay for this peptide reducing the amount of SnCl_2_ to a concentration of 1M, in which the total deprotection was achieved after 3 × 30 min treatment (Figure S11). This result suggested that the removal of the NO_2_ group could be sequence-dependent, thus potentially facilitating the use of even milder conditions than those generally proposed in some cases.

**Figure 6:**
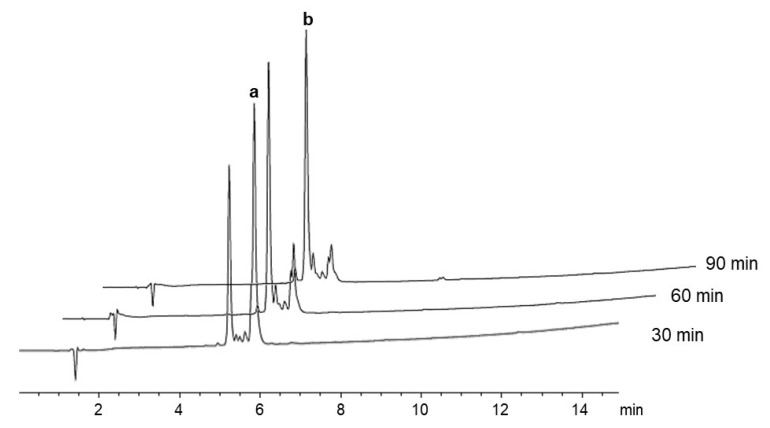
HPLC of the removal of the NO_2_ groups from protected bradykinin-NH-Rink-amide-resin using 2 M SnCl_2_-0.04 M phenol-0.2 M aq HCl in 2-MeTHF at 55 °C (MW). **a:** protected peptide; **b:** unprotected peptide. Elution: 5–95% of B into A in 15 min.

Next, these conditions were applied to remove the two NO_2_ groups of bradykinin. On this occasion, the kinetics was slower than in the preceding cases. In the search for alternatives to accelerate the reaction, two new assays were then run, one in an ultrasonic bath (Figure S12) and the other under microwave (MW) (Figure 6). Both rendered the expected results, namely a faster reaction, with ultrasound leading to total deprotection in 3 × 1 h treatments (as in the case of the LRF peptide) and MW in 3 × 30 min treatments. Although MW was faster, the ultrasonic bath may be a more accessible device.

**Figure 7:**
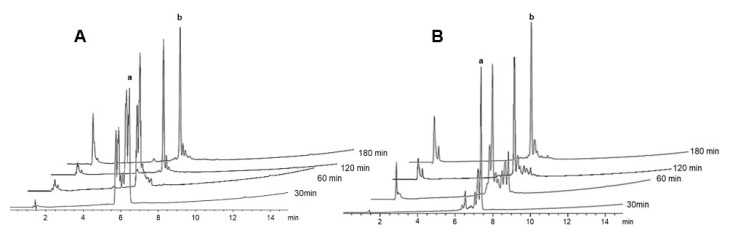
HPLC of the removal of the NO_2_ groups from H-[Trp(Boc)-Arg(NO_2_)]_2_-Pro-NH-Rink-amide resin (**A**) and H-[Trp(Boc)-Arg(NO_2_)]_3_-Pro-NH-Rink-amide resin (**B**) using 2 M SnCl_2_-0.04 M phenol-0.2 M aq HCl in 2-MeTHF at 55 °C. **a:** protected peptide; **b:** unprotected peptide. elution: 5-95% of B into A in 15 min.

Finally, to continue studying the removal of multiple NO_2_ groups, (RW)_n_P-NH_2_ (*n* = 2 or 3) were synthesized. The conditions applied were the general ones used previously under sonication. For both peptides, the results obtained after 3 × 1 h treatments were highly satisfactory and more than acceptable (Figure 7).

## 3. Conclusions

Arg is a key amino acid for the construction of peptides with relevant biological activity. Its guanidino side-chain requires protection to avoid side-reactions and to facilitate its solubility. Pbf, which is the most widely used protecting group, shows some drawbacks, mainly δ-lactam formation, stability to the TFA-based global deprotection conditions, and a high price, the latter due mainly to the preparation of the 2,2,4,6,7-pentamethyldihydrobenzofuran-5-sulfonyl chloride intermediate and its posterior incorporation into the guanidino group.

The NO_2_ group, which was first described by Bergmann, has been scarcely used in SPPS mainly because it has to be removed by catalytic hydrogenation in a post-cleavage step and this does not always provide satisfactory results. Furthermore, there has been some discrepancy in the literature regarding the potential of the NO_2_ group to suppress δ-lactam. Herein, we demonstrated that the electron-withdrawing effect of the NO_2_ group minimizes its nucleophilicity, which is translated into a reduction of the side-reaction when compared with Pbf protection. In contrast, bis-Boc protection is highly prone to δ-lactam formation. Furthermore, this group showed limited stability in DMF and in OxymaPure containing DMF, while the NO_2_ and the Pbf were stable.

Importantly, here we report on the development of a new and straightforward method for removing the NO_2_ group from Arg side chain while the protected peptide is still on the resin. This removal strategy is based on the use of SnCl_2_ as reducing agent in mild acid conditions using aq HCl as acid rectifier in a solution of 2-MeTHF, which is a green solvent, at 55 °C. As commonly found in peptide chemistry, we observed that the removal reaction could be sequence-dependent. However, in the cases in which the removal is not efficient, the concourse of sonochemistry significantly accelerates the removal reactions. The use of microwave heating also gave positive results. However, accessibility to this type of heating is limited in bioscience laboratories and therefore ultrasound baths emerge as the method of choice. In some demanding sequences involving the unstable Trp residue, the appearance of small satellite peaks could be attributable to side-reactions, which should be further studied for each case. Although currently the price of the Fmoc-Arg(NO_2_)-OH is, in a 25-g scale, slightly more expensive than Fmoc-Arg(Pbf)-OH, in large-scale production the former should be more economical due to it using less expensive raw materials.

Overall, in terms of effectiveness and handling, Fmoc-Arg(NO_2_)-OH emerges as an interesting alternative to the existing Arg derivatives.

## 4. Materials and Methods

### 4.1. Materials

All reagents and solvents were from commercial suppliers and were used without further purification. Fmoc amino acids and Fmoc-Rink-amide AM PS resin (loading 0.74 mmol/g) were purchased from Iris Biotech GMBH (Marktredwitz, Germany). DIC and OxymaPure were a gift from Luxembourg Biotech. (Ness Ziona, Israel) and *N*,*N*-diidsopropylethylamine (DIEA), SnCl_2_, and piperidine were supplied by Sigma-Aldrich (St. Louis, MO, USA). Organic solvents (DMF, CH_2_Cl_2_ (DCM)) and HPLC quality acetonitrile (CH_3_CN) were purchased from Merck (Kenilworth, NJ, USA). Milli-Q water was used for RP-HPLC analyses. Microwave treatments were carried using a Discover SP (CEM, Matthews, NC, USA). Scientech Ultrasonic Cleaner (Labotec, Durban, South Africa) was used for the ultrasound treatment. Analytical HPLC was performed on an Agilent 1100 system using a Phenomenex Aeris^TM^C18 (3.6 μm, 4.6 × 150 mm) column, with flow rate of 1.0 mL/min and UV detection at 220 nm. Chemstation software was used for data processing. Buffer A: 0.1% TFA in H_2_O; buffer B: 0.1% TFA in CH_3_CN. LC-MS was performed on Ultimate™ 3000, Aeris^TM^ 3.6 µm Wide pore column, Phenomenex C18 (150 × 4.6 mm) column.

### 4.2. Peptide Synthesis

The peptides used in this study were synthesized manually using a syringe fitted with a porous polyethylene disc and attached to a vacuum trap for easy filtration. Synthesis were carried out 0.1 or 0.2 mmol scale Rink-amide-PS resin (loading = 0.74 mmol/g) using Fmoc/tBu protocols. For Fmoc removal, 20% of piperidine in DMF was used. Couplings were performed by 1.5 eq. of an equimolar mixture of Fmoc-amino acid derivative, DIC and Oxyma in DMF or NBP, which was allowed to react for 1 h. Global deprotection and cleavage was performed by adding TFA-TIS-H_2_O (95:2.5:2.5) to the peptidyl-resin for 1 h at rt. Then, chilled ether was added to precipitate the peptides and after centrifugation and decantation, the peptides were taken up in water. The crudes obtained were analyzed by HPLC and finally were lyophilized. The peptide identities were confirmed by LC-MS.

### 4.3. Stability Study Procedure

Solutions (0.2 M) of each protected Arg analogue in the absence or presence of OxymaPure (0.2 M) were prepared and kept in sealed vials. Aliquots (10 µL) at different times of each one were taken and diluted with CH_3_CN (490 µL), and 1µL of this mixture was injected in the HPLC.

### 4.4. δ-Lactam Formation

An equimolar solution of Fmoc-Arg(X)-OH/DIC/OxymaPure (1.5 equiv.) in DMF or NBP was added to the tripeptidyl resin (H-GFL-resin) at 45 °C and kept reacting for 2 h. A 10-μL aliquot of the supernatant was taken from the mixture at 0, 30, 60, and 120 min and diluted to 0.5 mL with CH_3_CN. From this solution, 1 μL was injected and analyzed by RP-HPLC (30–95% B into A in 15 min). After 120 min, Fmoc was removed using 20% piperidine in DMF, and peptides were cleaved from the resin as described before. Amino acid coupling was quantified by integration of the peaks obtained in their HPLC (10–25% B into A in 15 min).

### 4.5. Removal of the NO_2_ Group

The removal solution was prepared by taking SnCl_2_ (1.8 g), phenol (4 mg) in 2-MeTHF (2 mL), then aqHCl (98 µL) was added and the mixture was sonicated until total solubility of SnCl_2_. The final volume was then made up to 5 mL using 2-MeTHF, which gave a 0.2 N concentration of HCl.

Peptide resin (50 mg) was washed with a solution of 2-MeTHF-HClaq (0.1%) (1.5 mL, 2 × 1 min). After filtration, the SnCl_2_ solution was added (1.5 mL) and the syringe was sealed. The reaction was then left for 30 min at 55 °C with sporadic stirring. The solution was filtered off and the resin was washed with 2-MeTHF. An aliquot was then taken for mini cleavage using TFA-TIS-H_2_O (95:2.5:2.5) (60 µL) as before. Fresh removal solution was added to the peptide resin and the process was repeated.

## Figures and Tables

**Table 1:** Stability of Fmoc-Arg(X)-OH in DMF (*N,N-*dimethylformamide) and NBP (*N-*butylpyrrolidone) at room temperature.

	0 h	1 h	24 h	48 h	10 d	15 d	20 d	30 d
**Fmoc-Arg(Boc)_2_-OH, DMF**	88.8	88.6	86.9	85.0	77.6	65.1	58.5	51.2
**Fmoc-Arg(Boc)_2_-OH, NBP**	88.8	88.4	85.8	83.5	71.8	62.0	52.2	37.7
**Fmoc-Arg(NO_2_)-OH** **(DMF or NBP) ^1^**	100	100	100	100	100			
**Fmoc-Arg(Pbf)-OH** **(DMF or NBP) ^1^**	100	100	100	100	100			

^1^ The study was stopped after 10 days, no changes were observed during this period.

## Supplementary Materials

The supplementary information is available online at https://www.mdpi.com/1422-0067/21/12/4464/s1.
