# Supplementary material for: Revisiting NO2 as Protecting Group of Arginine in Solid-Phase Peptide Synthesis
Source: Int J Mol Sci. 2020 Jun 23;21(12):4464. doi: 10.3390/ijms21124464 (PMC7352207; doi:10.3390/ijms21124464)

Supporting Information

Revising NO_2 as Protecting Group of Arginine in Solid-Phase Peptide synthesis

Mahama Alhassan,[1#] Ashish Kumar,[1,2#] John Lopez[3], Fernando Albericio,[1,4,5*] Beatriz G. de la Torre[2*]

[#] Both authors contributed equally

[1]Peptide Science Laboratory, School of Chemistry and Physics, University of KwaZulu-Natal, Durban 4001, South Africa; [2]KwaZulu-Natal Research Innovation and Sequencing Platform (KRISP), School of Laboratory Medicine and Medical Sciences, College of Health Sciences, University of KwaZulu-Natal, Durban 4041, South Africa; [3]Novartis Pharma AG, Lichtstrasse 35, CH-4056 Basel, Switzerland; [4]CIBER-BBN, Networking Centre on Bioengineering, Biomaterials and Nanomedicine, Barcelona Science Park, University of Barcelona, 08028 Barcelona, Spain; [5]Department of Organic Chemistry, University of Barcelona, 08028 Barcelona, Spain

Table S1. Summary of all trial used in the removal of NO2 group from the peptides.

#	$SnCl_2$ (M)	Phenol (M)	Acid (M)	Solvent	T °C	Time (h)	Peptide	Removal (%)
1	8	0.04	AcOH (0.016)	DMF	55	2	LRF	43.2
2	8	0.04	HCl-Dioxane (0.064)	DMF	55	2	LRF	39.6
3	8	0.04	HCl-Dioxane (0.064)	DCM/DMF	55	8	LRF	100.0
4	8	0.04	HCl-Dioxane (0.2)	MeOH	RT	2	LRF	2.9
5	8	0.04	HCl-Dioxane (0.2)	EtOH	55	8	LRF	98.3
6	8	0.04	HCl-Dioxane (0.064)	NBP	55	2	LRF	43.1
7	8	0.04	HCl-Dioxane (0.2)	NBP	55	2	LRF	66.7
8	8	0.04	HCl-Dioxane (0.064)	2-MeTHF	55	2	LRF	98.1
9	8	0.04	HCl-Dioxane (0.2)	2-MeTHF	55	2	LRF	98.4
10	2	0.04	HCl-Dioxane (0.064)	2-MeTHF	55	2	LRF	76.7
11	2	0.04	HCl-Dioxane (0.064)	CPME	55	2	LRF	37.1
12	2	0.04	HCl-Dioxane (0.2)	CPME	55	2	LRF	34.6
13	2	0.04	aq HCl (0.2)	2-MeTHF	55	2	LRF	98.0
14	2	-	aq HCl (0.2)	2-MeTHF	55	2	LRF	91.0
15	2	0.04	aq HCl (0.2)*	2-MeTHF	55	2	LRF	100.0
16	2	0.04	aq HCl (0.2)*	2-MeTHF	55	1	RGD	100.0
17	1	0.04	aq HCl (0.2)*	2-MeTHF	55	2.5	LRF	100.0
18	1	0.04	aq HCl (0.2)*	2-MeTHF	55	1.5	RGD	100.0
19	1	0.04	aq HCl (0.2)*	2-MeTHF	40	2	RGD	42.6
20	2	0.04	aq HCl (0.2)*	2-MeTHF	55	1.5	Bradykinin	26.1
21	2	0.04	aq HCl (0.2)*/ultrasound	2-MeTHF	55	3	Bradykinin	94.0
22	2	0.04	aq HCl (0.2)*/microwave	2-MeTHF	55	1.5	Bradykinin	92.5
23	2	0.04	aq HCl (0.2)*/ultrasound	2-MeTHF	55	3	$(RW)_2P$	100
24	2	0.04	aq HCl (0.2)*/ultrasound	2-MeTHF	55	3	$(RW)_3P$	100

Figure S1: Stability of Fmoc-Arg(Boc)$_2$-OH at room temperature in DMF. Elution: 30-95% of B into A in 15 min. Mobile phase A: 0.1% TFA in H_2O; mobile phase B: 0.1% TFA in CH_3CN; λ = 220 nm; Phenomenex Aeris^{TM}C18 (3.6 μm, 4.6 × 150 mm) column

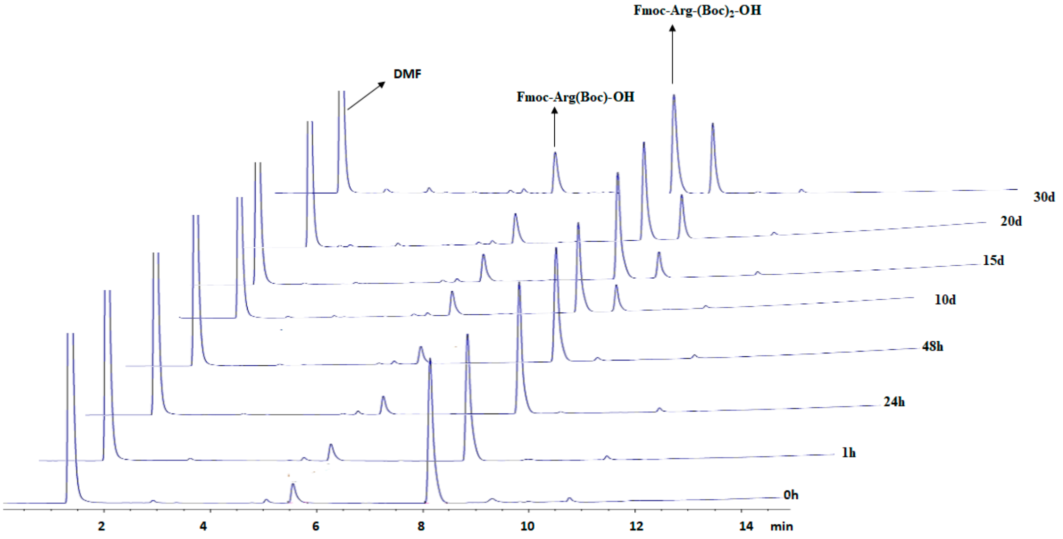

Figure S2: Stability of Fmoc-Arg(Boc)$_2$-OH at room temperature in NBP. Elution: 30-95% of B into A in 15 min. Mobile phase A: 0.1% TFA in H_2O; mobile phase B: 0.1% TFA in CH_3CN; λ = 220 nm; Phenomenex Aeris^{TM}C18 (3.6 μm, 4.6 × 150 mm) column.

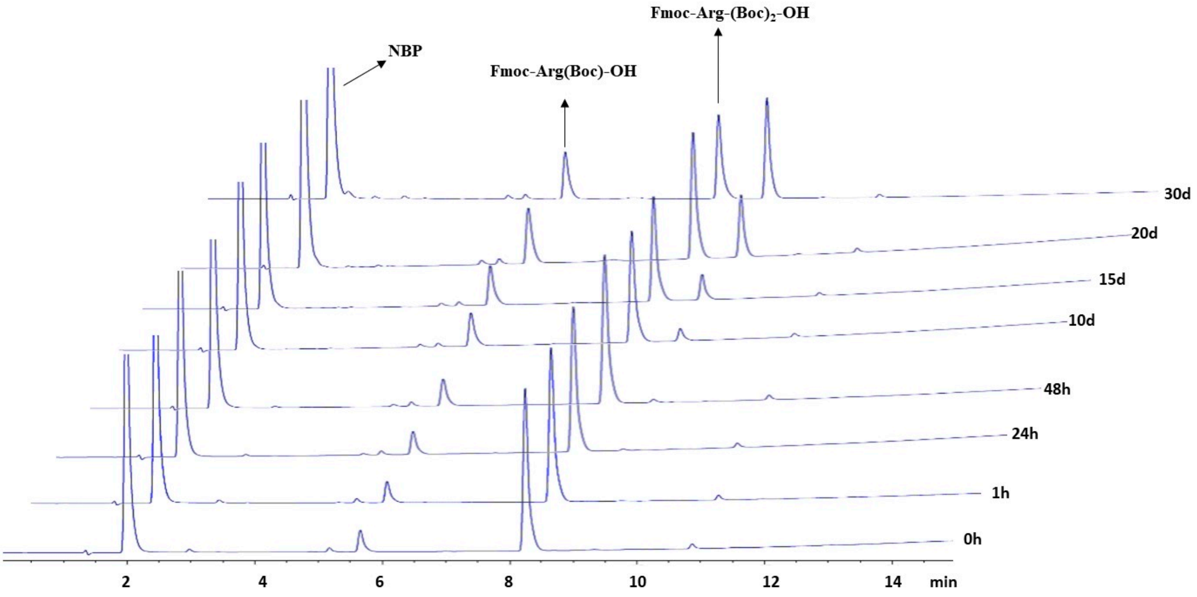

Figure S3: Stability of Fmoc-Arg(NO_2)-OH at room temperature in DMF. Elution: 30-95% of B into A in 15 min. Mobile phase A: 0.1% TFA in H_2O; mobile phase B: 0.1% TFA in CH_3CN; λ = 220 nm; Phenomenex Aeris^{TM}C18 (3.6 μm, 4.6 × 150 mm) column.

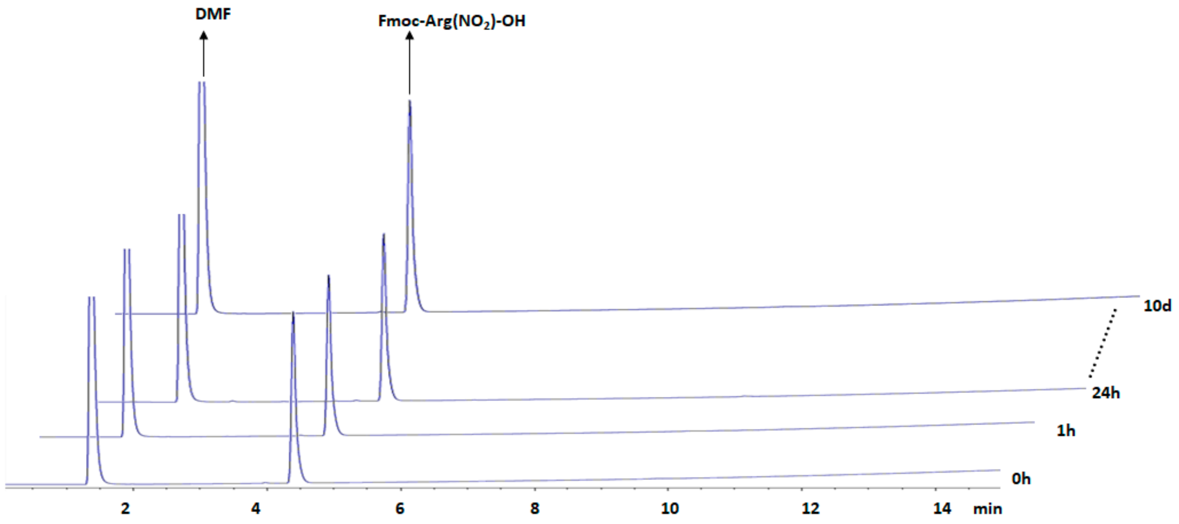

Figure S4: Stability of Fmoc-Arg(NO_2)-OH at room temperature in NBP. Elution: 30-95% of B into A in 15 min. Mobile phase A: 0.1% TFA in H_2O; mobile phase B: 0.1% TFA in CH_3CN; λ = 220 nm; Phenomenex Aeris^{TM}C18 (3.6 µm, 4.6 × 150 mm) column.

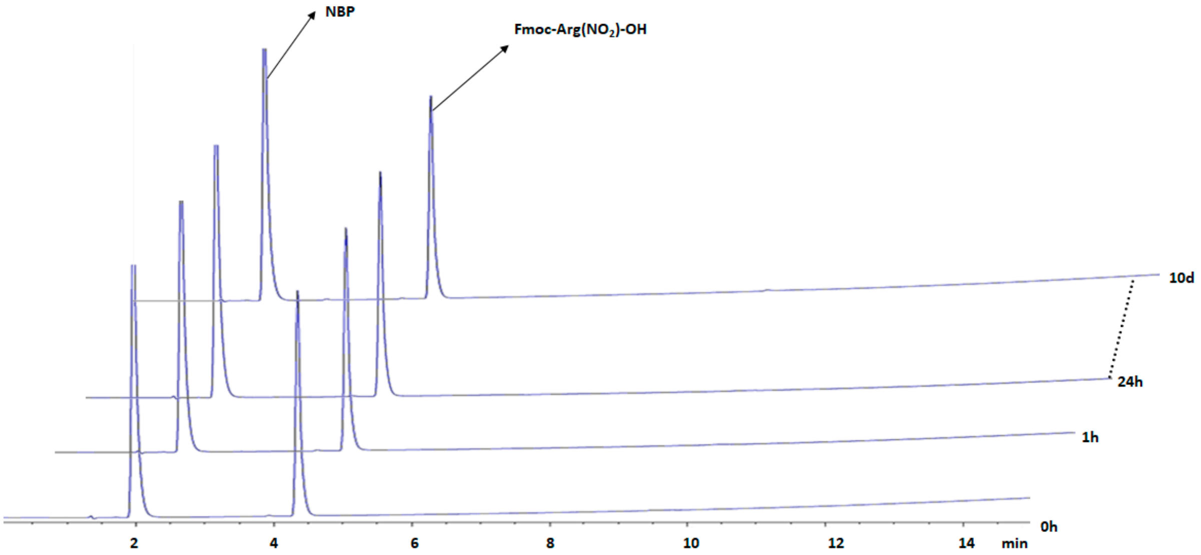

Figure S5: Stability of Fmoc-Arg(Pbf)-OH at room temperature in DMF. Elution: 30-95% of B into A in 15 min. Mobile phase A: 0.1% TFA in H_2O; mobile phase B: 0.1% TFA in CH_3CN; λ = 220 nm; Phenomenex Aeris^{TM}C18 (3.6 µm, 4.6 × 150 mm) column.

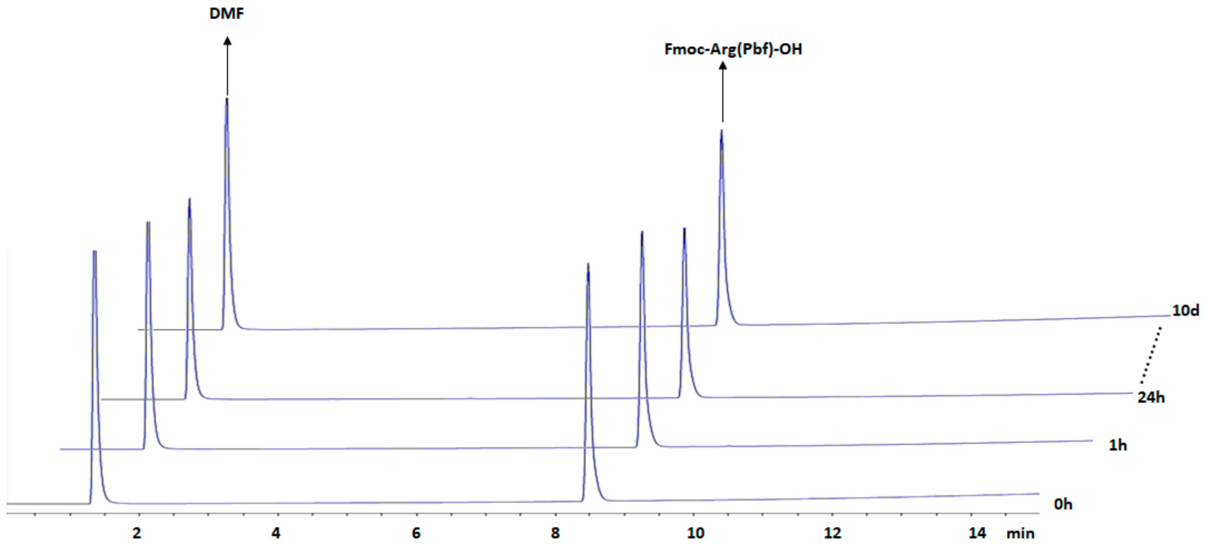

Figure S6: Stability of Fmoc-Arg(Pbf)-OH at room temperature in NBP. Elution: 30-95% of B into A in 15 min. Mobile phase A: 0.1% TFA in H_2O; mobile phase B: 0.1% TFA in CH_3CN; λ = 220 nm; Phenomenex Aeris™C18 (3.6 µm, 4.6 × 150 mm) column.

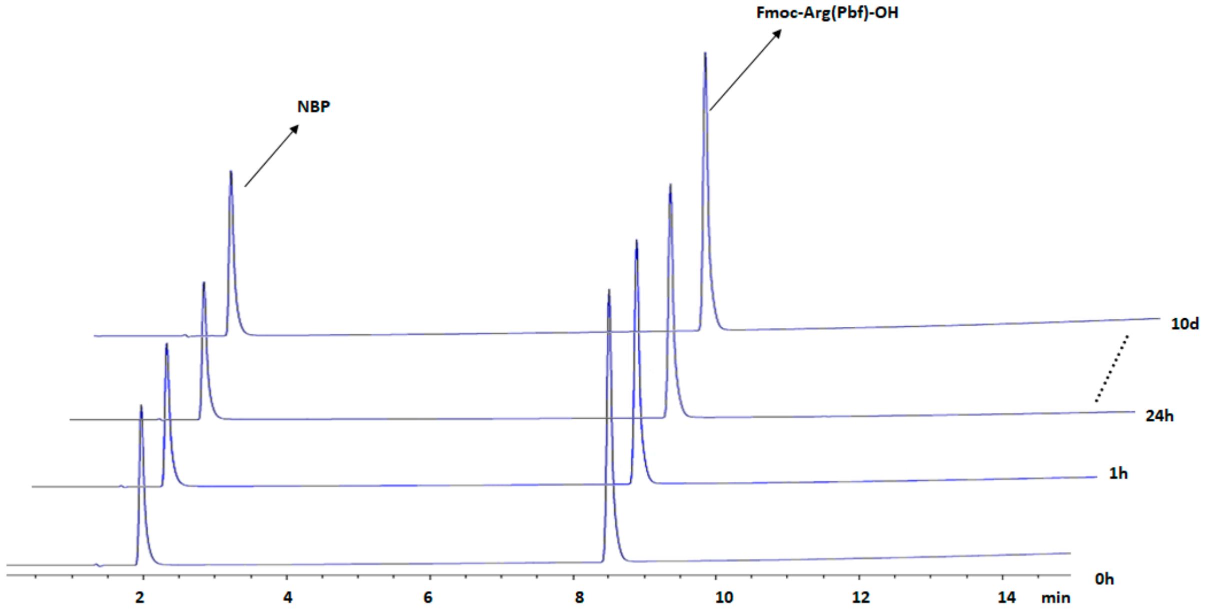

Figure S7: Stability of Fmoc-Arg(NO_2)-OH with OxymaPure at 45°C in DMF Elution: 30-95% of B into A in 15 min. Mobile phase A: 0.1% TFA in H_2O; mobile phase B: 0.1% TFA in CH_3CN; λ = 220 nm; Phenomenex Aeris™C18 (3.6 µm, 4.6 × 150 mm) column.

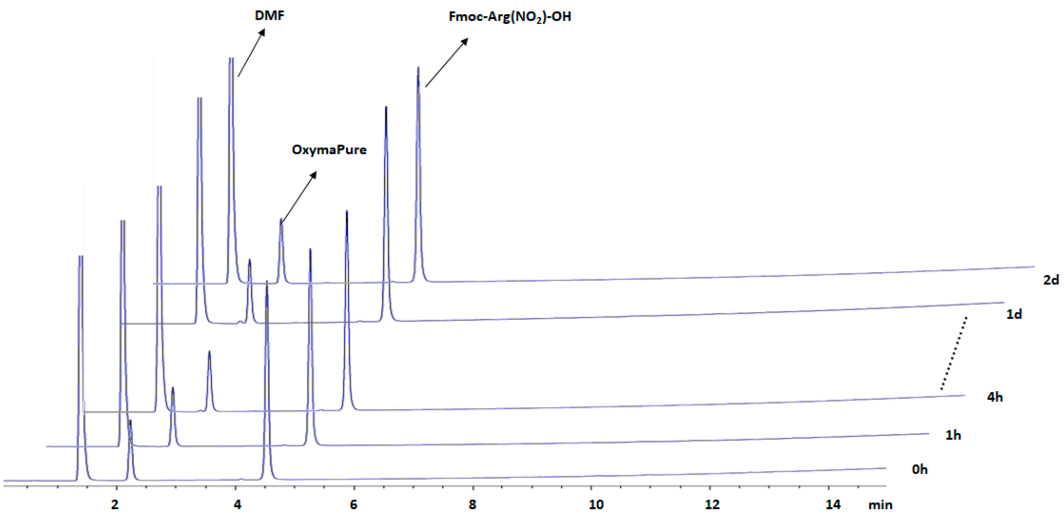

Figure S8: Stability of Fmoc-Arg(NO_2)-OH with OxymaPure at 45°C in NBP Elution: 30-95% of B into A in 15 min. Mobile phase A: 0.1% TFA in H_2O; mobile phase B: 0.1% TFA in CH_3CN; λ = 220 nm; Phenomenex Aeris™C18 (3.6 µm, 4.6 × 150 mm) column.

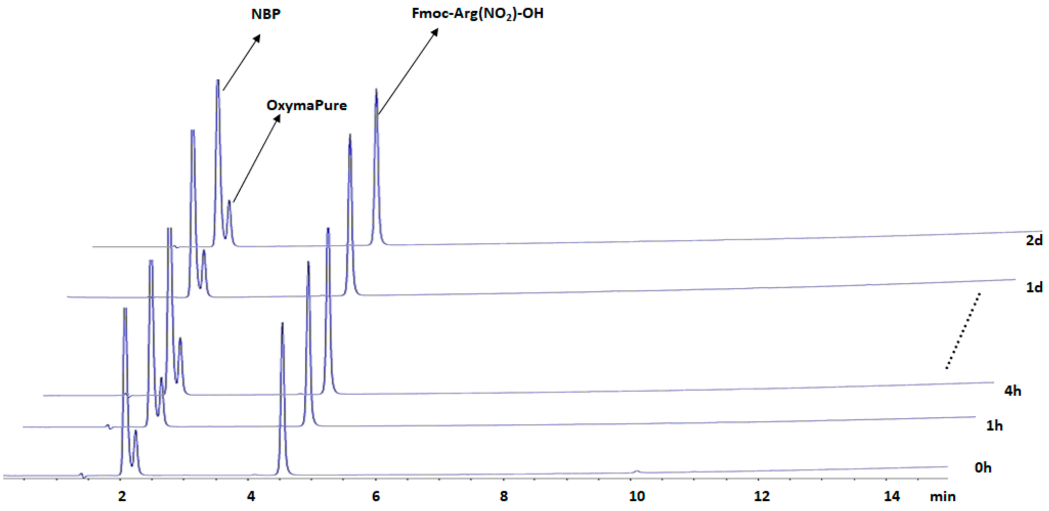

Figure S9: Stability of Fmoc-Arg(Pbf)-OH with OxymaPure at 45°C in DMF. Elution: 30-95% of B into A in 15 min. Mobile phase A: 0.1% TFA in H_2O; mobile phase B: 0.1% TFA in CH_3CN; λ = 220 nm; Phenomenex Aeris™C18 (3.6 µm, 4.6 × 150 mm) column..

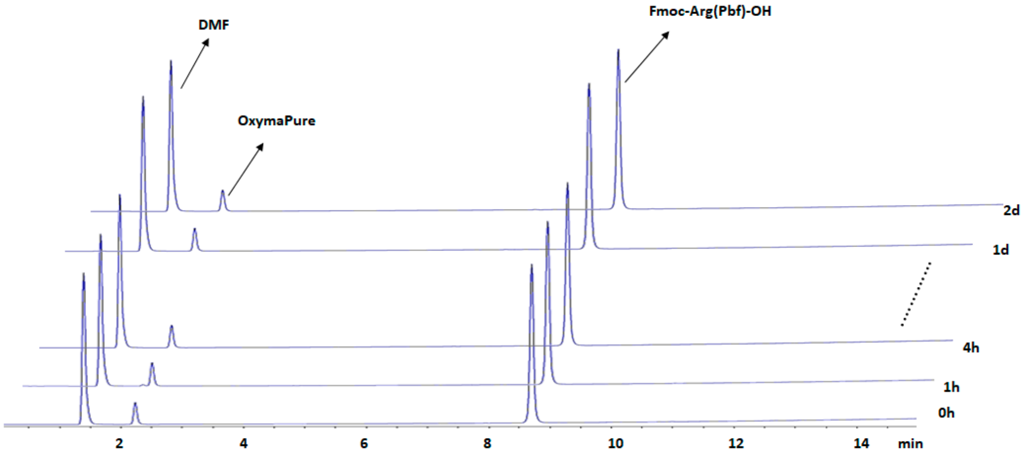

Figure S10: Stability of Fmoc-Arg(Pbf)-OH with OxymaPure at 45°C in NBP. Elution: 30-95% of B into A in 15 min. Mobile phase A: 0.1% TFA in H_2O; mobile phase B: 0.1% TFA in CH_3CN; λ = 220 nm; Phenomenex Aeris™C18 (3.6 µm, 4.6 × 150 mm) column.

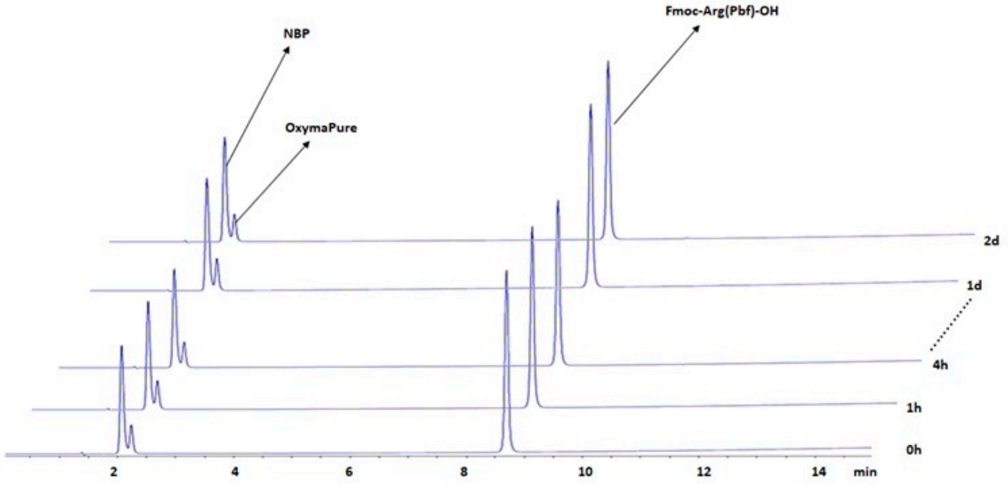

Figure S11: The removal of the NO_2 group from H-Asp(OtBu)-Phe-Gly-Arg(NO_2)-Gly-NH-Rink amide-resin using 1M $SnCl_2$, 0.04MPhenol in 2-MeTHF-0.2N HCl at 55°C. Elution: 10-25% of B into A in 15 min. Mobile phase A: 0.1% TFA in H_2O; mobile phase B: 0.1% TFA in CH_3CN; λ = 220 nm; Phenomenex Aeris^{TM}C18 (3.6 µm, 4.6 × 150 mm) column.

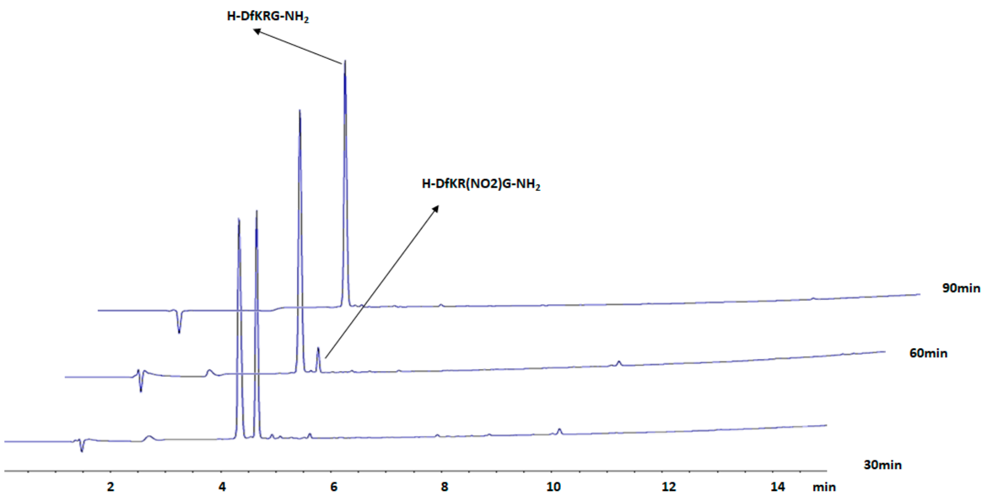

Figure S12: The removal of the NO_2 groups from protected bradykinin-NH-Rink amide-resin using 2M$SnCl_2$ 0.04MPhenol in 2-MeTHF acidify with 0.2N HClaq at 55°C in ultrasonic bath. Elution: 5-95% of B into A in 15 min. Mobile phase A: 0.1% TFA in H_2O; mobile phase B: 0.1% TFA in CH_3CN; λ = 220 nm; Phenomenex Aeris^{TM}C18 (3.6 µm, 4.6 × 150 mm) column.

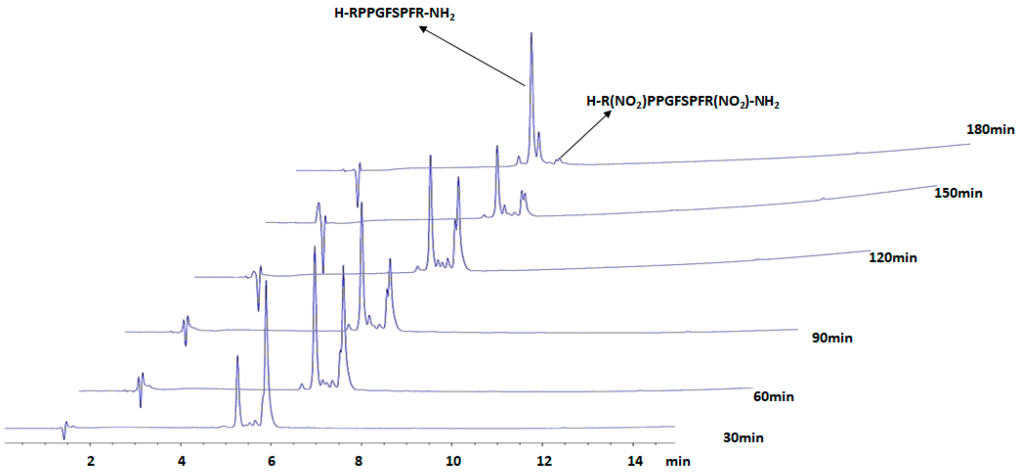

Supplement: Supplementary file 1 [file ijms-21-04464-s001.pdf]
